# Mineral patterns in hair: A decisive factor between reproducible and repeat breeder dairy cows

**DOI:** 10.1371/journal.pone.0301362

**Published:** 2024-04-02

**Authors:** Hyun-Joo Lim, Seunghoon Lee, Woncheoul Park, Eungwoo Park, Jae Gyu Yoo

**Affiliations:** National Institute of Animal Science, Rural Development Administration, Wanju-gun, Jeollabuk-Do, Republic of Korea; Bangladesh Agricultural University, BANGLADESH

## Abstract

Reproduction, especially impregnation, is a critical aspect of dairy cow management that directly influences herd milk productivity. We conducted a noninvasive hair mineral assay to compare the mineral profiles of two dairy cow groups: reproducible and repeat breeder, by investigating the levels of 11 essential minerals (Ca, Mg, Na, K, Fe, Cu, Mn, Zn, Cr, Se, and P) and 6 toxic elements (Hg, Pb, Cd, Al, As, and Ni) in both groups. We also conducted principal component and correlation matrix analyses to compare hair mineral patterns between the groups. Compared to their reproducible counterparts, repeat breeder cows had lower levels of Na, K, and Se. However, Fe, Cd, Al, and As levels were higher in repeat breeders than in their reproducible counterparts. The correlation matrix showed notable correlation patterns for each group. Ca, K, and Na levels were positively correlated in reproducible cows, whereas repeat breeder cows showed positive correlations only between Ca and K levels. Se showed positive correlations with Zn only in the reproducible cow group. Negative correlations were not found in the reproducible group, whereas the repeat breeder group exhibited 7 negative correlations. Despite the limitations of hair mineral analysis, this study provided useful insights into the reproductive potential of dairy cows. These findings aid in easing the prediction of repeat breeder occurrences in herds and are expected to facilitate timely mineral supplementation and other interventions to improve overall herd reproduction in dairy farms.

## Introduction

Minerals, as naturally occurring inorganic chemical compounds abundant on Earth are essential components of animal physiology, maintaining animals’ overall health and well-being. Minerals are the foundational components of the body and play pivotal roles in various physiological processes, such as cell replication and differentiation, as well as in activating enzymes and hormone functions. They are classified as macro and trace minerals. Macro minerals, such as calcium, phosphorus, sodium, potassium, and magnesium, are required in relatively large amounts by the body and play essential roles in physiological functions [1]. Trace minerals, such as copper, zinc, iron, iodine, manganese, and chromium, are required in smaller amounts by the body but are also important for animal health [2]. However, certain amounts of toxic elements including arsenic, mercury, cadmium, and lead, adversely affect the body [3].

In farm animals, mineral supplementation is crucial for appropriate mineral balance and preserving the optimal health of a livestock herd [4], thereby enhancing farm management efficiency and providing economic benefits to farms. Mineral imbalance causes various problems in dairy cattle breeding, including retained fetal membranes and weak calf syndrome, resulting in lowered reproductive efficiency [5, 6]. Among these reproductive issues, repeat breeding is a noteworthy reproductive disorder in dairy cattle that manifests as infertility even after three or more inseminations without the presence of infections or anatomical abnormalities [7]. The reproductive disruptions resulting from dairy cattle breeding repetitively can be ascribed to various factors, including ovarian dynamics, hormone pattern interaction, and the stimulation of estrus behavior. These factors have the potential to escalate insemination expenses and diminish milk yield [8].

In general, reproductive events, such as services per conception, the postpartum interval, calf survival, and the calving interval, are correlated with the body condition score (BCS) [9]. The incidence of repeat breeding among lactating dairy cows varies depending on the herd management style as well as the environment type and specific region. The reported incidence of repeat breeding in the dairy cow population is approximately 10% [8, 10]. However, the primary origin of repeat breeding is unclear and multifactorial; therefore, preventing the dysfunction of reproductive hormones or enzyme release is important to reduce repeat breeding in herds. One potential approach in ensuring good health of entire herds is the periodic assessment of mineral status.

The mineral status of animals can be examined using invasive or noninvasive methods. The invasive methods include liver and serum analyses. Although mineral status analysis from the liver is the best method to reflect overall body mineral status, this method is difficult to conduct as it requires either biopsies from live animals or sampling from dead animals [11, 12]. Additionally, because blood analysis is involved, the blood collection procedure may cause stress in animals [13]. Notably, compared with the analysis of plasma, feces, urine, and other tissues, hair analysis is noninvasive and offers longer-term information regarding trace mineral accumulation [14, 15]. Hair composition is closely related to blood composition and concentration, as hair follicles are connected to the dermis and function as miniature organs comprising blood vessels, sebaceous sweat glands, and nerves [16]. In recent decades, human hair analysis has been used to estimate the levels of trace minerals and toxic elements in groups and individuals [17–20], as hair is a fundamental biological specimen that is convenient to collect, transport, and store [21, 22]. In animals, hair samples collected from dogs [23], horses [24], reindeer [25], and cows [26–28] were recently used to investigate the correlations between trace minerals and immune responses, health conditions, and diseases.

In the present study, we examined hair samples from cows that consistently became pregnant after artificial insemination against those from cows that repeatedly reproduced. We aimed to determine the correlations and disparities between the two groups by examining hair-derived mineral levels. Additionally, we elicited novel, specific patterns for mineral balances only in repeated breeders through correlation matrix analysis. The results of this study indicate that assessing the mineral status of the hair of repeated breeders could serve as a practical marker to determine the timing of mineral supplementation in the management of cattle at the herd and individual levels, thereby addressing health and reproductive problems.

## Materials and methods

### Selection of animals and herd management

All animal care methods and experiments were conducted in accordance with the "Guide for the Care and Use of Laboratory Animals" established by the National Institute of Animal Science (Approval number 2015–125). All dairy cows which used in this study are Holstein breed (scientific name: *Bos Taurus*). The average age of the observed cows was 66 ± 22 months and the parity ranged from 0 to 6. Eighteen cows that have normal estrus cyclesbut failed to conceive more than five times even after successful artificial inseminations (AIs) were classified as ‘repeat breeder cows’. Pregnant cows and those with functional anestrus or cystic ovaries on rectal palpation were excluded from being categorized as repeat breeder cows. Eighteen dairy cows conceived after AI were selected as normal, ‘reproducible control cows’. The cows were housed in a building with 10–20 animals per straw-bedded pen. The cows were maintained in free stalls, and were fed standard rations (TMR, total mixed ration), including concentrates (40.8%), corn (cracked) (8.3%), full-fat soybeans (4.6%), soluble dried grains (2.1%), corn silage (11.7%), Italian ryegrass silage (30.0%), and supplements (2.5%; limestone, sodium bicarbonate, salts, vitamins, and minerals). Chemical analysis on the basis of dry matter(DM) showed that the TMR contained 44.45%DM moisture, 6.97%DM crude protein, 2.52%DM ether extract, 12.19%DM crude fibre, 3.25%DM crude ash, 14.57%DM acid detergent fibre, and 16.05%DM neutral detergent fibre. Water access was freely available at all times to facilitate food intake.

### Reproductive management

Cows were checked for estrus twice daily: once in the morning and once in the evening. The cows were considered in estrus when they stood for mounting by another cow. In addition, cows were observed for physical (vulvar edema and mucus discharge) and behavioral changes in the reproductive tract. Estrus was diagnosed based on the absence of a corpus luteum and the presence of a large fluctuating follicle on rectal palpation. Cows expressing signs of true estrus were inseminated 12 h after the onset of estrus. The AI and its schedules were recorded using an inseminator. AI was performed using frozen-thawed semen in a 0.25-mL straw containing at least 10 million motile spermatozoa from a single bull with proven fertility. Frozen semen was thawed in a 30–37°C water bath for 30 s. Cows were inseminated by the deposition of semen into the uterine body. A pregnancy diagnosis was confirmed 60 days post-insemination based on rectal palpation or ultrasonic examination.

### Body condition scoring (BCS)

BCS was determined on a scale of 1 to 5 in 0.25 increments for all dairy cattle. The assessment was conducted through visual and tactile appraisal along the animal’s top line from the loin to the tail head area, including the hips, pin bones, and tail head [29].

### Sampling and laboratory analysis

Hair samples were collected from the shoulders of the cows, and 100 mg of each hair sample was weighed and placed in a 50-mL polypropylene tube (DigiTube; SCP Science, Québec, Canada). To prevent contamination, the shoulder area, un-soiled by feces was selected as the sampling site. The blade of the electronic hair clipper was sterilized with 70% alcohol before each sampling. Owing to ongoing monitoring of the mineral status of hair, the entire hair, including the proximal, distal, and middle sections, was examined. For the hair mineral assay, ICP-MS (7700X; Agilent Technologies, Santa Clara, CA, USA) was performed with the assistance of GunSei Biotech Inc. (Gwacheon, Republic of Korea; http://www.gunseibio.com).

### Statistical and visualization analysis

Statistical analyses were performed using GraphPad Prism 5 software (GraphPad, San Diego, CA, USA). We used a one-way analysis of variance (ANOVA) test after arcsine transformation of the proportional data to analyze significant differences. The principal component analysis (PCA) and correlation plots were visualized using the R packages “ggfortify” and “Corrplot”, respectively. Two investigators participated in blind statistical and visualization analysis.

## Results

### General characteristics of repeat breeder and reproducible dairy cows

Ultrasonographic examination revealed no detectable clinical reproductive disorders, and the corresponding normal physiological structures were detected during various estrous cycle stages. Although these differences were not statistically significant, repeat breeding dairy cattle tended to be older and had lower parity than reproducible cows, as Table 1 illustrates. It was found that reproducible cows needed fewer AI services than repeat breeder cows (*p* = 0.0001). The mean BCS of repeat breeders was significantly higher than that of the reproducible cows (*p* = 0.001).

**Table 1:** General characteristics of repeat breeder and reproducible dairy cows.

	Repeat breeder cows^a^	Reproducible cows^a^	*p*-value
**Age of cows (months)**	69.78 ± 3.92	61.22 ± 6.06	0.244
**Parity**	1.39 ± 0.3	2.22 ± 0.33	0.072
**No. of artificial insemination**	8.5 ± 0.69	1.39 ± 0.11	0.0001
**Body condition score (BCS)**	3.71 ± 0.11	3.15 ± 0.86	0.001

^a^Mean±SEM

### Hair mineral analysis

This study examined 11 essential minerals and 6 toxic elements in hair. The 11 essential minerals comprised five macro-minerals (Ca, Mg, Na, K, and P) and six trace minerals (Fe, Cu, Mn, Zn, Cr, and Se). The 6 toxic elements were Hg, Pb, Cd, Al, As, and Ni. The analysis results of the 11 minerals with detected levels sufficient to compare reproducible and repeat breeder cows are presented in Table 2. Repeat breeder cows had mean hair concentrations of Na, K, and Se that were significantly lower than reproducible cows (all *p* < 0.01). In contrast, the mean hair Fe concentration in repeat breeder cows was significantly higher than that in reproducible cows (*p* = 0.0103). There was no discernible difference in the concentrations of Ca, Mg, P, Cu, Mn, Zn, and Cr between reproducible cows and repeat breeder cows. Replicable cows had significantly lower levels of Cd, Al, and As than reproducible cows when it came to toxic minerals (all *p* < 0.05; Table 3). No significant differences between the groups were observed in Hg, Pb, or Ni concentrations (Table 3).

**Table 2:** Hair mineral levels in repeat breeder and reproducible dairy cows.

Element	Repeat breeder cows (ppm)^a^	Reproducible cows (ppm)^a^	*p*-value
**Ca**	666.62 ± 203.9	706.42 ± 234.2	0.8987
**Mg**	719.48 ± 75.75	908.06 ± 81.29	0.0988
**Na**	1121.21 ± 391.5	2777.17 ± 398.2	0.0055
**K**	2856.41 ± 289.6	4196.44 ± 363.1	0.0067
**P**	349.84 ± 20.1	392.12 ± 26.28	0.2099
**Fe**	357.03 ± 73.82	140.41 ± 30.1	0.0103
**Cu**	9.75 ± 1.65	9.65 ± 0.65	0.9538
**Mn**	42.12 ± 4.42	30.69 ± 4.69	0.0856
**Zn**	510.02 ± 112.6	550.52 ± 68.43	0.7605
**Cr**	0.99 ± 0.3	0.54 ± 0.09	0.1651
**Se**	0.59 ± 0.05	0.81 ± 0.06	0.0092

^a^Mean±SEM

**Table 3:** Concentration of hair toxic elements in repeat breeder and reproducible dairy cows.

Element	Repeat breeder cows (ppm)^a^	Reproducible cows (ppm)^a^	*p*-value
**Hg**	0.045 ± 0.01	0.15 ± 0.08	0.2163
**Pb**	1.975 ± 0.53	1.25 ± 0.15	0.1957
**Cd**	0.04 ± 0.006	0.02 ± 0.002	0.0024
**Al**	241.14 ± 64.16	64.79 ± 15.83	0.0116
**As**	0.17 ± 0.02	0.11 ± 0.01	0.0149
**Ni**	0.39 ± 0.05	0.28 ± 0.03	0.0887

^a^Mean±*SEM*

### Correlation of minerals in repeat breeder and reproducible dairy cows

**Fig 1:**
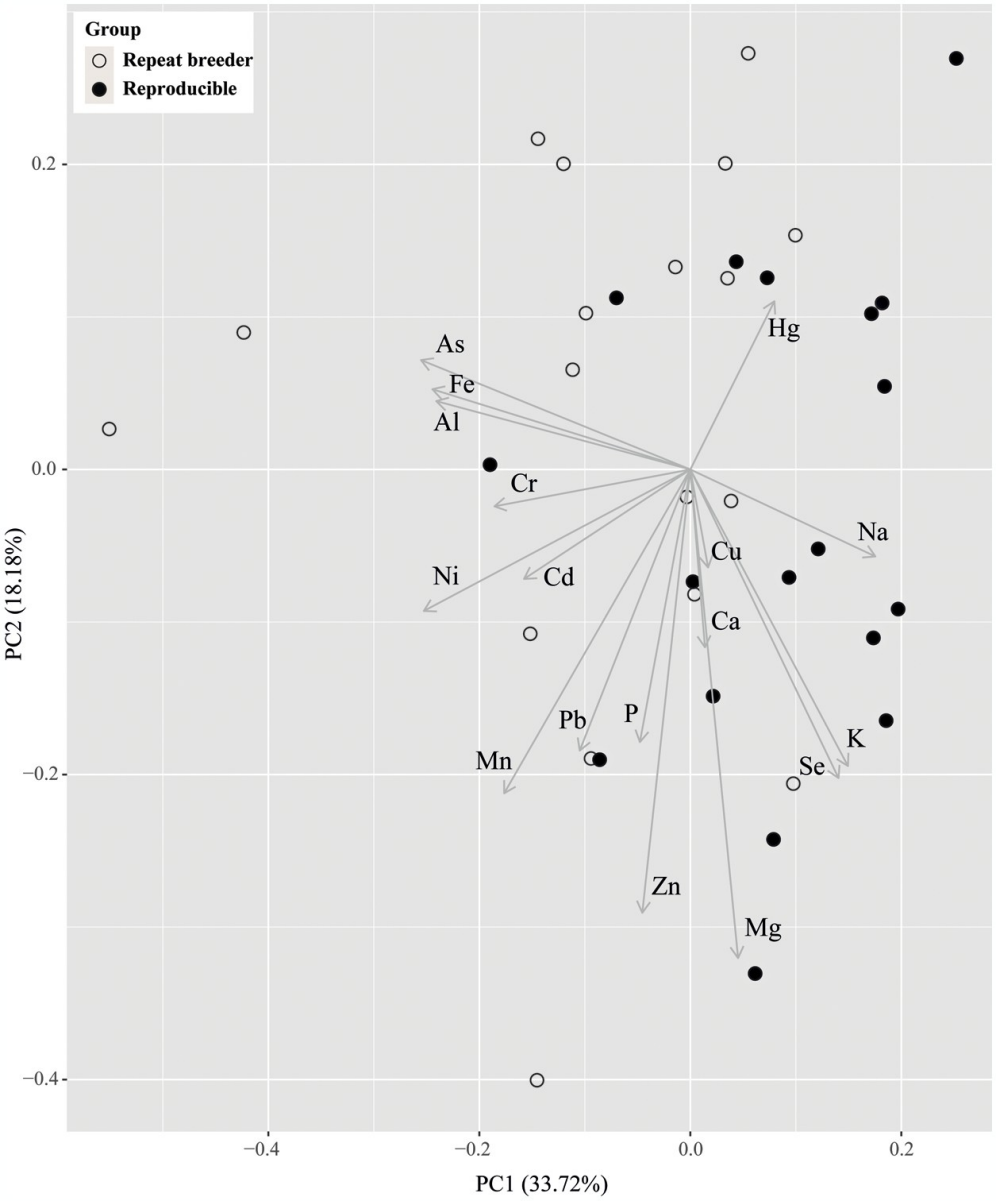
Principal component analysis (PCA) of repeat breeder and reproducible dairy cows.

As shown in Fig 1, PCA was conducted to confirm which principal minerals showed differences between repeat breeders and reproducible dairy cows. PC1 contributed 33.72%, and PC2 contributed 18.18% to the total component variation in the PCA plot. Most repeat breeder individuals were on the negative side, whereas most reproducible individuals were on the positive side based on the zero point of PC1. This separation between the reproducible and repeat breeder groups was mostly affected by Na, K, and Se on the positive side and As, Al, and Fe on the negative side.

PC1 represents the first principal component, and PC2 is the second principal component. Arrows on the plot correspond to individual minerals or elements. White dots indicate individuals of repeat breeder dairy cows, and black dots represent individuals from reproducible dairy cows.

**Fig 2:**
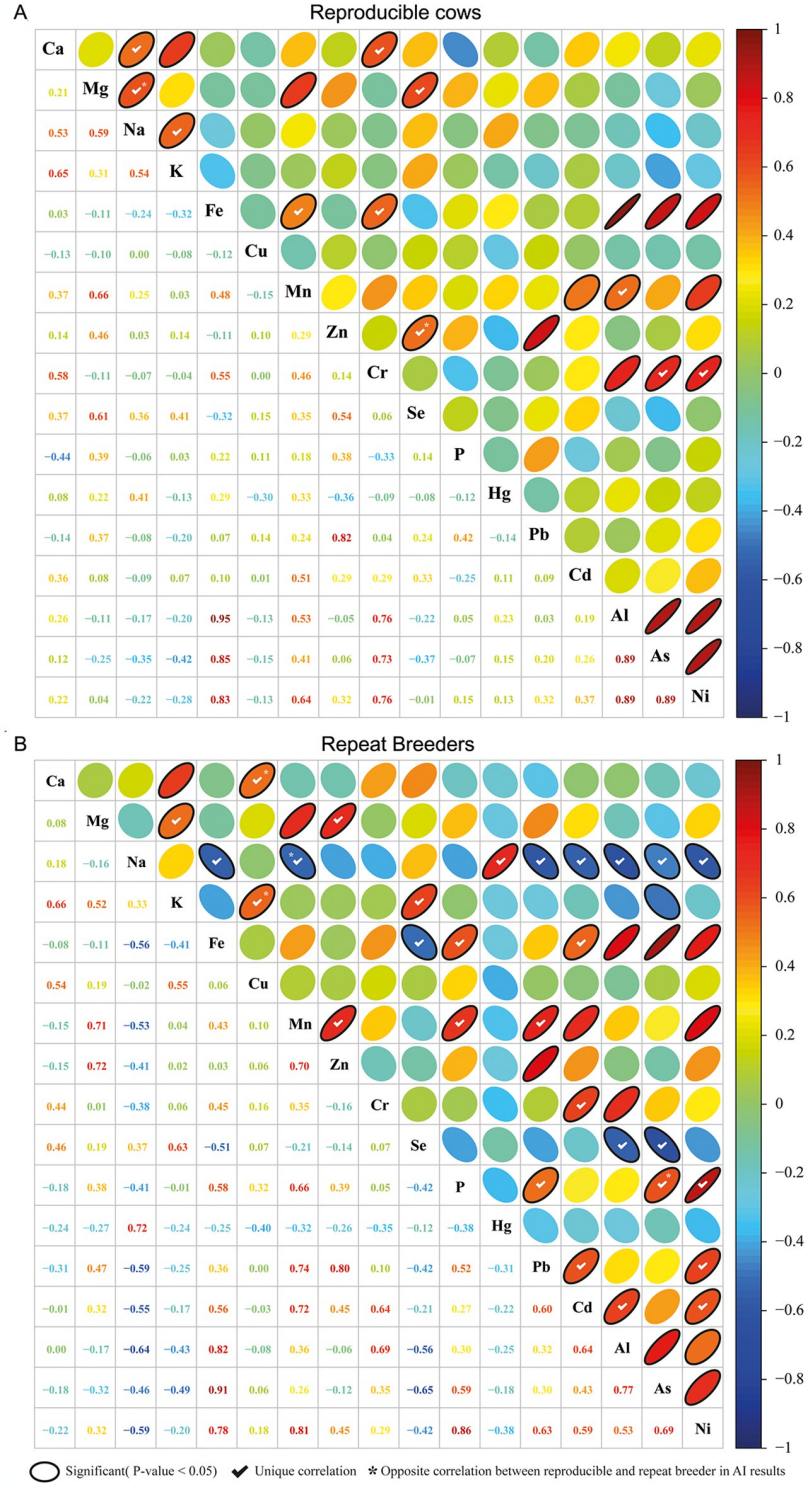
Correlation matrix analysis of 11 essential minerals and six toxic elements. (A) Correlation of minerals in reproducible dairy cow group. (B) Correlation of minerals in repeat breeder dairy cow group. The red series color indicates positive correlation and the blue series color indicates negative correlation at the intersection between two minerals. An elliptical outline indicate significance (*p* < 0.05) between two minerals. Check mark means that unique significant correlation between two minerals is showed at only a group between reproducible and repeat breeders. Asterisk mark is used when the positive or negative correlation between two minerals in a group changes to opposite significant correlation in other group.

The correlation matrix shows the relationships between the two minerals in each repeat breeder and reproducible cow group (Fig 2). The shades of red at the cross points between the two minerals illustrate a positive correlation, whereas the shades of blue side represents a negative correlation in each group. In both correlation matrices of reproducible and repeat breeder cows, significant correlations (*p* < 0.05) are indicated by an oval shape, unique correlations between both groups are represented by a checkmark shape, and the opposite correlation between both groups is denoted by a star shape.

As shown in Fig 2A, the reproducible dairy cow group exhibited 10 significantly positive correlations among the essential minerals; of these, the correlations between Na-K and Zn-Se were notable. The repeat breeder dairy cow groups showed 3 significant negative correlations and 10 significant positive correlations among the essential minerals (Fig 2B). We observed significant negative correlations between Na and Fe, Na and Mn, and Fe and Se only in the repeat breeder group. Unlike the reproducible group, no significant correlations were identified between Na-K and Zn-Se in the repeat breeder group.

Among the 6 toxic element correlations, we identified only 3 and 7 significantly positive correlations in the reproducible and repeat breeder dairy cow groups, respectively. Aluminum was significantly positively correlated with As and Ni in both the groups. Unlike the reproducible dairy cow group, the repeat breeder dairy cow group displayed significantly positive correlations between Cd-Al and Cd-Ni. Overall, the number of positive correlations among the toxic elements in the repeat breeder group was higher than that in the reproducible group.

The correlation between essential minerals and toxic elements showed that the reproducible dairy cow group had 10 significant positive correlations, whereas the repeat breeder dairy cow group had 14 significantly positive correlations and 8 significant negative correlations. Notably, Na was significantly negatively correlated with Pb, Cd, Al, As, and Ni in the repeat breeder group. Similarly, Se showed a significantly negative correlation with Al and As in the repeat breeder group.

## Discussion

Repeat breeding in dairy cows is caused by many factors, including inappropriate herd management, inadequate estrus detection, semen quality, insemination techniques, malformation of reproductive organs, endocrine disorders, reproductive system diseases, early embryonic death, lower parity, and the BCS [30–37]. However, generalizing the predominant causes of repeat breeding is challenging owing to significant individual variance. A multifactorial problem involving several extrinsic and intrinsic factors coupled with an individual animal could also be a cause. One possible solution to address repeat breeding is to maintain better health conditions through mineral management [4]. Mineral imbalances in the body can lead to various health problems through impediments to cellular events, including immune response and hormone release from cells [38]. Consequently, repeat breeders are likely to suffer from a temporary endocrine imbalance, resulting in ovulation failure, fertilization failure, or early embryonic loss [39–41]. Therefore, periodic monitoring of the mineral status of a herd is important to ensure the control of herd mineral balance.

The present study applied hair mineral analysis, a noninvasive sampling method for the assessment of herd mineral status. Although the BCS is also a good noninvasive indicator of reproductive performance, hair mineral analysis could be a more reliable indicator. Mineral analysis provides invariable objective results from hair samples; however, the BCS tends to vary depending on the subjectivity of examiners. In this study, we compared the hair mineral patterns of repeat breeder cows to those of reproducible cows and explored the potential mineral-related factors that showed different patterns between the two dairy cow groups. Neither group exhibited significant differences in parity or age, whereas the repeat breeder dairy cow group had a significantly higher BCS. Although both groups received the same feed composition, higher BCS (>3.5.) was only measured in the repeat breeder dairy cow group. BCS values of <3.0 or >3.5 are strongly associated with reduced reproductive performance in dairy cows [42]; however, we primarily aimed to investigate the hair mineral characteristics of repeat breeder dairy cows rather than focusing on finding a direct relationship between BCS and repeat breeding.

The present study revealed statistically significant differences between the two groups. Specifically, differences were noted within the essential mineral category (Na, K, Fe, and Se) as well as in the toxic elements (Cd, Al, and As). Sodium, K, and Se levels were significantly higher in the reproducible dairy cow group except for Fe, whereas Cd, Al, and As levels were significantly higher in the repeat breeder group. Further, PCA also revealed differences in the four essential minerals and three toxic elements. The National Academies of Sciences, Engineering, and Medicine are among the organizations that determine the daily minimum requirements of essential minerals based on species. It should be noted that body and hair mineral statuses do not match in the same units; therefore, estimating whether essential minerals are deficient or excessive in the body based solely on the absolute values of hair minerals is not feasible [11]. However, we can infer that repeat breeders tend to have relatively lower accumulations of essential minerals and higher accumulations of toxic elements in their bodies.

Sodium is strongly associated with the regulation of cellular homeostasis, body fluid balance, electrolyte equilibrium, and blood pressure [43]. Since the adrenal cortical hormone aldosterone is essential in controlling sodium retention, lower sodium levels in animals may be a sign of adrenal dysfunction [44]. Adrenal function is closely linked to pregnancy through alterations in the hypothalamic-pituitary-adrenal axis [45]. We observed that Na levels were lower in repeat breeder cows, with positive correlations only between Ca and K. However, the reproducible cow group showed positive correlations among all three elements (Ca, K, and Na). Specifically, repeat breeders did not show a Na-K correlation. The balance between Na^+^ and K^+^ is vital for membrane potential development, hormone secretion, and cardiac function [46, 47]. The membrane protein of the sodium-potassium pump maintains the concentration of Na^+^ and K^+^ in the animal body through the primary active transport of ions. This pump protein is essential for blastocoel formation and blastocyst hatching in animal embryos [48]. Therefore, our results for repeat breeders, which showed a low Na level and a disrupted correlation between Na and K, while reproducible cows showed a positive correlation between Na and K, could be an indicator of adverse reproductive effects, such as repeat breeding.

Selenium is an essential component of glutathione peroxidase, which protects the cellular lipid membranes against oxidative damage [49]. Antioxidants are beneficial to female reproduction [50]. In addition, Se deficiency is related to poor growth, health, and reproductive problems such as weak and irregular estrus, poor uterine involution, abortion, and cystic ovaries [51–53]. Selenium is a well-known mineral related to reproductive performance in dairy cows, as Se supplementation improves conception rates and reduces the incidence of placental retention and cystic ovaries [54]. In our study, Se levels in repeat breeders were significantly lower than those in reproducible cows. Notably, we confirmed that the positive correlation between Se and Zn in the reproducible group was not observed in the repeat breeder group. Zinc also functions as an antioxidant and has a synergistic antioxidant effect with Se [55]. Therefore, in the case of a disrupted correlation between Se and Zn in hair minerals, simultaneously supplementing Se and Zn in the herd is recommended to increase antioxidant levels. Regarding negative correlations in the two groups, Na did not show any correlations with other minerals and toxic elements in the reproducible dairy cow group, whereas the repeat breeder dairy cow group exhibited seven negative correlations with Na. These correlations in repeat breeders may have been caused by the higher levels of Fe and toxic elements. The higher levels of Fe and toxic elements in the repeat breeders group may have also led to a positive correlation. Similar to the negative correlation between Na and toxic elements, trace mineral Se also exhibited two negative correlations, only in repeat breeder cows. Overall, when examining the correlation matrix, essential minerals in repeat breeder cows tended to show more negative correlations with toxic elements than those in the reproducible dairy cows. In addition, the repeat breeder group exhibited higher levels of Fe than the reproducible group. Although Fe is essential for hemoglobin synthesis and oxygen transportation in red blood cells, excess Fe can be toxic [56]. Therefore, higher Fe levels, similar to toxic elements in repeat breeders, may negatively affect reproductive performance. Consequently, hair Fe and other toxic elements could serve as indicators of excessive accumulation in the animal body.

In this study, we explored the relationship between distinctive elements in the hair mineral status and their function or reproductive ability. Regarding the differences in mineral values between sampling areas, we have previously verified that there were no significant differences (*p* < 0.05) in the mineral values of the hair samples from each area (data not shown). However, it is important to note that hair mineral patterns may differ from the golden standard for body mineral status, liver mineral analysis, and may also vary from patterns observed in blood and hair mineral analyses [21]. When it comes to the Zn status in bone and muscle samples, which better represent the primary Zn pools than liver sample, even liver mineral analysis is not ideal at reflecting body mineral state [4]. In order to ascertain which source of mineral analysis most accurately depicts the body’s mineral status for various elements, more thorough investigation will be needed in the future. In the present study, we presented repeat breeder hair mineral patterns in a herd of dairy cows by conducting hair mineral analyses and referring the function of minerals. These results can be used to assess the likelihood of repeat breeders occurring in dairy cow herds and help in enhancing the reproductive capacity and overall health of the herd through on- time mineral supplementation.

## Supporting information

S1 Table All abbreviations in manuscript. (DOCX)
