# Supplementary material for: Mineral patterns in hair: A decisive factor between reproducible and repeat breeder dairy cows
Source: PLoS One. 2024 Apr 2;19(4):e0301362. doi: 10.1371/journal.pone.0301362 (PMC10986949; doi:10.1371/journal.pone.0301362)
Supplement: S1 Table — (DOCX) [file pone.0301362.s001.docx]

**S1 table. All abbreviations in manuscript.**

| **Abbreviation** | **Full name** |
| --- | --- |
| **AI** | **Artificial Insemination** |
| **Al** | **Aluminum** |
| **As** | **Arsenic** |
| **BCS** | **Body Condition Score** |
| **Ca** | **Calcium** |
| **Cd** | **Cadmium** |
| **Cr** | **Chromium** |
| **Cu** | **Copper** |
| **DM** | **Dry Matter** |
| **Fe** | **Iron** |
| **Hg** | **Mercury** |
| **K** | **Potassium** |
| **Mg** | **Magnesium** |
| **Mn** | **Manganese** |
| **Na** | **Sodium** |
| **Ni** | **Nickel** |
| **P** | **Phosphorus** |
| **Pb** | **Lead** |
| **PCA** | **Principal Component Analysis** |
| **PC1** | **Principal Component 1** |
| **PC2** | **Principal Component 2** |
| **Se** | **Selenium** |
| **TMR** | **Total Mixed Ration** |
| **Zn** | **Zinc** |
